# Shedding light on stellate cells

**DOI:** 10.7554/eLife.41041

**Published:** 2018-09-14

**Authors:** Andrew S Alexander, Michael E Hasselmo

**Affiliations:** Department of Psychological and Brain SciencesBoston UniversityBostonUnited States

**Keywords:** entorhinal cortex, stellate cells, grid cells, spatial navigation, memory, hippocampus, Mouse

## Abstract

The relationship between grid cells and two types of neurons found in the medial entorhinal cortex has been clarified.

**Related research article** Rowland DC, Obenhaus HA, Skytoen ER, Zhang Q, Kentros CG, Moser EI, Moser MB. 2018. Functional properties of stellate cells in medial entorhinal cortex layer II. *eLife* **7**:e36664. doi: 10.7554/eLife.36664

Most people can remember the floorplan of their current home and the layout of their local supermarket. They might also be able to create a virtual map of their current location, their home and the supermarket, which allows them to mentally navigate from one place to the next. Our sense of location depends on a network of regions in the brain, including the hippocampus and its neighbor, the medial entorhinal cortex (MEC).

The different types of neurons within these structures work together to form a sort of inbuilt GPS that tracks our position relative to other objects or places in the environment. In the hippocampus, place cells are activated when an animal occupies a single position in the environment (O'Keefe and Dostrovsky, 1971). In the MEC, head direction cells and border cells become active when an animal faces a particular direction or is near a border (Sargolini et al., 2006; Solstad et al., 2008). The MEC also contains grid cells that – much like the black squares on a chess board – represent multiple equally-spaced locations in an environment via their firing patterns (Hafting et al., 2005).

Previous research has shown that the inputs of the MEC into the hippocampus – in particular from the grid cells – are potentially crucial for the spatial and memory functions (Schlesiger et al., 2015). Grid cells reside predominantly in an area of the MEC known as layer II, where two morphologically distinct sub-populations of neurons, the stellate and pyramidal cells, exist.

Both stellate and pyramidal cells have different physiological properties and connect to the hippocampus through different pathways (Alonso and Klink, 1993). Stellate cells form a prominent connection directly into multiple sub-regions of the hippocampus, while the density of the connections between the pyramidal neurons and the hippocampus is significantly less. However, the exact role of stellate and pyramidal cells has so far remained unclear.

Several studies have reported that both stellate and pyramidal cells could be grid cells, while others found that the proportion of grid cells within the stellate sub-population was virtually nonexistent (Domnisoru et al., 2013; Schmidt-Hieber and Häusser, 2013; Tang et al., 2014). Thus, it has remained unclear whether MEC neurons that exhibit grid firing or other spatial responses belong to the sub-class of MEC layer II cells that do indeed project into the hippocampus.

Now, in eLife, May-Britt Moser and colleagues at the Norwegian University of Science and Technology – including David Rowland as first author – report new insights into these cells (Rowland et al., 2018). Using sophisticated genetic tools paired with electrical recordings from single neurons in free-moving mice, they could assess the relationship between stellate and pyramidal cell sub-populations, and other known spatial coding neurons within the MEC layer II.

Rowland et al. used a technique called optogenetics, in which genetically modified neurons that produce light-sensitive proteins can either be activated or silenced with light. The mouse model used in the experiments expressed a light-sensitive protein called ArchT in the stellate cells of layer II, which meant that these neurons could be shut off by exposing them to light of a specific wavelength.

Rowland et al. measured the activity of layer II neurons while the mice freely explored an open space. Then, the same neurons were recorded while simultaneously exposed to light (a process referred to as ‘phototagging’). All cells that were silenced within moments of the light onset were ‘tagged’ as layer II stellate cells. This allowed a comparison of firing properties during the free-foraging session between the tagged stellate neurons and untagged populations composed primarily of pyramidal cells.

The results showed that the tagged stellate cell population had similar, if not stronger, spatial firing properties compared to the untagged cell population. Grid cells existed in similar numbers in both the tagged stellate and untagged populations. This suggests that the stellate cells projecting into the hippocampus include cells from a range of functional cell types and thus, may help the hippocampus to process information about location.

The work of Rowland et al. has resolved discrepancies between previous reports and brought to light important questions. For example, how do stellate grid cells, pyramidal grid cells and other types of spatial cells shape spatial processing and memory, and are there any differences between them? To what degree do these morphologically distinct, yet functionally overlapping, sub-populations depend on one another? These questions aside, the latest work demonstrates the power of phototagging as a means to better characterize circuit-specific projections within the brain regions that support navigation.
